# The correlation between tumor-associated macrophage infiltration and progression in cervical carcinoma

**DOI:** 10.1042/BSR20203145

**Published:** 2021-05-20

**Authors:** Fan Guo, Weina Kong, Gang Zhao, Zhenzhen Cheng, Le Ai, Jie Lv, Yangchun Feng, Xiumin Ma

**Affiliations:** 1 State Key Laboratory of Pathogenesis, Prevention and Treatment of High Incidence Diseases in Central Asia, Clinical Laboratory Center, Tumor Hospital Affiliated to Xinjiang Medical University, Urumqi 830011, China; 2 The First Affiliated Hospital of Xinjiang Medical University, Urumqi 830054, China; 3 Department of Blood transfusion, Affiliated Traditional Chinese Medicine Hospital of Xinjiang Medical University, Urumqi 830000, China

**Keywords:** Cervical cancer, Clinicopathological features, Meta-analysis, Progression, Tumor-associated macrophage (TAM)

## Abstract

Tumor microenvironment (TME) plays a particularly important role in the progression, invasion and metastasis of cervical carcinoma (CC). Tumor-associated macrophages (TAMs) are significant components of the tumor microenvironment in CC. However, the results of studies on the correlation between TAMs and progression in CC are still controversial. This research aimed to investigate the relationship between TAMs infiltration and progression in CC. A total of 100 patients with CC were included in the study. The correlation between TAMs and clinicopathologic features was studied. Besides, a systematic literature search was conducted from legitimate electronic databases to specifically evaluate the role of TAMs in TME of cervical carcinoma. In the meta-analysis, high stromal CD68^+^ TAMs density was relevant to lymph node metastasis (WMD = 11.89, 95% CI: 5.30–18.47). At the same time, CD163^+^ M2 TAM density was associated with lymph node metastasis (OR = 2.42, 95% CI: 1.09–5.37; WMD = 39.37, 95% CI: 28.25–50.49) and FIGO stage (WMD = -33.60, 95% CI: -45.04 to -22.16). This was further confirmed in the experimental study of 100 tissues of cervical cancer. It supported a critical role of TAMs as a prospective predictor of cervical cancer. In conclusion, CD68^+^ TAM and CD163^+^ M2 TAM infiltration in CC were associated with tumor progression. And CD163^+^ M2 TAM infiltration was associated with more advanced FIGO stage and lymph node metastasis in CC.

## Introduction

Cervical carcinoma (CC) is one of the most common gynecological tumors [1]. Although relevant results have been achieved in the field of primary and secondary prevention, it continues to be the second leading cause of cancer deaths in young women, causing 9 people in the 20–39 years group to die per week in developed countries such as the United States [2]. Over the past decade, the proportion of patients with locally advanced diseases among CC cases has remained stable in most developed countries [3]. Immunotherapy, as a new method of tumor therapy, is being popularized in clinic. Tumor-associated macrophages (TAMs) are an important part of the tumor microenvironment (TME) [4,5]. They act as immune regulators in TME and are potential targets for immunotherapy of cancer patients [6].

One of the most specific characteristics of TAMs is their plasticity and heterogeneity. According to the stimulation of TME, TAM can be divided into two main types: ‘M1-like’ (classically activated) and ‘M2 -like’ (alternatively activated). M1-like polarized TAM is considered to have proinflammatory and antitumor effects, while M2-like has immunosuppressive and protumor effects [7–9]. The research on the correlation between TAMs and CC has been a hot spot in recent years. The change of TME leads to the alternative polarization of macrophages and transformation in biological functions, which plays an important role in the development of CC.

The dual role of TAM in tumor progression has been supported by studies in different tumor models *in vitro* and *in vivo*. Current studies have shown that the characteristic molecular marker of TAMs is CD68, while M2-like polarized TAMs are commonly used CD163 [10,11], CD23 [12], and CD204 [13]. However, the role of TAMs in the progression of CC is still controversial. Therefore, we conducted a study to validate the association between TAMs and clinicopathological parameters in CC and then performed a meta-analysis to evaluate the role of different types of TAMs in the TME of CC by pooling data from 11 eligible studies.

## Materials and methods

### Sample collection

A total of 100 cases of surgical resection specimens (cancer and paracarcinoma or normal tissue) were acquired from the Department of Pathology, the third Clinical Medical College of Xinjiang Medical University (affiliated tumor Hospital, Urumqi, Xinjiang). Samples were collected from January 2017 to July 2019. All specimens were fixed with 10% formaldehyde and embedded in paraffin.

We collected clinical features while collecting tumor paraffin specimens. Cases who received chemotherapy or radiotherapy before surgery were excluded. Each paraffin specimen included two pathological tissue sections. All the cases of study were confirmed to be CC by surgery and pathology. The patient data were evaluated according to the recommendations of the International Federation of Obstetrics and Gynecology (FIGO) revised in 2018. All procedures followed the ethical standards of the institution and the National Human Experimentation Committee and comply with the ‘Helsinki Declaration’ of 1964 and later. The Ethics Committee of the Third Clinical Medical College of Xinjiang Medical University approved the study and consent procedures. All patients in the study obtained informed consent or alternative consent.

### Immunohistochemistry (IHC) and immunohistochemical evaluation

Rabbit anti-CD68 antibody was purchased from Boster Biological Technology (diluted 1:50, BA3638, California, UK), and rabbit anti-CD163 antibody was bought from Bioss Biotechnology Co. Ltd. (diluted 1:200, bs-2527R, Beijing, China). H&E staining was used to assess the pathological slices. The detection method was immunohistochemistry. All slices were produced using the following procedures: dewaxing, rehydration, xylene, ethanol washing, steamed classification, boiling water washing, dissolving endogenous peroxide physical activity by incubation with 3% H_2_O_2_, antigen repair, staining and so on. Immunohistochemical staining was manual. Phosphate buffer saline (PBS) instead of the primary antibody was the negative control in the experiment, and the previous positive one was the positive control. Computer-aided image analysis and ImageJ software were used to calculate the positive staining. Two independent pathologists who did not know the clinical data evaluated the immunostaining and averaged the results.

Macrophage infiltration was quantitatively estimated in stroma of CC. The positive expression of CD68 and CD163 antibodies were brown in cytoplasm. Each sample was screened at a low magnification (×100), and five ‘hot spots' were selected for further analysis. The average macrophage count (per mm^2^) in these five regions of each slide was estimated at a high power (×400) magnification rate. The density of CD68- and CD163- positive cells were calculated as the positive staining area divided by the total analytical areas. For the five ’hot spots’, the average value was used to represent the final macrophage density for each slice.

### Heterogeneity and reproducibility

The heterogeneity between pairwise sections from the same patient was modest, as we observed that a paired *t*-test correlations of the density of CD68- or CD163- positive macrophages was 0.65 and 0.54, respectively. There was no significant difference between the samples (*P*=0.09). Furthermore, we randomly selected 25% of the glass tablets and scored twice in order to confirm reproducibility. And we evaluated all the results in a similar way.

### Search strategy

The case–control studies published about the macrophage infiltration and association with clinicopathogenic features in the tissues of cervical cancer were electronically retrieved in PubMed, Cochrane Library, Embase, Web of Science, CNKI, WanFang Data, Google and Google Scholar on June, 2020. Studies published between January 1996 and June 2020 were selected using the following searching strategies: ‘cervical cancer’ or ‘cervical carcinoma’ or ‘uterine cervix cancer’ and ‘tumor-associated macrophages' or ‘tumor infiltrating macrophages' or ‘macrophage cells' or ’macrophage’ or ‘myeloid cells’. The last retrieval time was June 20, 2020. The references of the identified articles were also scanned to find and identify potentially eligible articles.

### Inclusion and exclusion criteria

The inclusion criteria for our systematic review were: (i) papers that published in domestic or foreign journals included a case–control study on the relationship between macrophage infiltration and clinicopathologic features of cervical cancer; (ii) all patients had complete clinical and pathological data, without undergoing radiochemotherapy or other antitumor treatment before sampling, and the stage of CC was made according to the FIGO recommendations; (iii) control group was normal cervical tissue or adjacent tumor tissue; (iv) the method of detecting macrophage infiltration was the same, IHC; (v) macrophage density or their number was evaluated in the studies; (vi) macrophage infiltration in CC was described as high (above the cut-off value or positive) and low (below the cut-off value or negative) density; (vii) domestic literatures must be published in the national core journals, and foreign ones were published in full-text English. If the publications used different macrophage markers to study the same group of patients, all of them were included for marker-specific analysis.

The exclusion criteria were as follows: (i) conference abstracts, case reports, reviews, *in vitro* or animal studies and non-English/non-Chinese publications; (ii) duplication of reports and poor quality of studies; (iii) blind methods were not used to assess immunohistochemical results, i.e., pathologists were unaware of the patient’s pathology and clinical status; (iv) inconsistent criteria for assessing immunohistochemistry results (the mean macrophage count of five areas in each case was estimated according to the proportion of positively stained cells).

### Literature selection and data extraction

Two researchers (F.G. and Y.C.F.) independently reviewed and evaluated the all-included studies by using the Newcastle-–Ottawa Scale (NOS) [14]. The disagreement between the researchers was resolved by the third author (G.Z.). After reviewing the full text, two investigators (W.N.K. and Z.Z.C.) independently extracted data from eligible studies. The following information was extracted from each article: surname of the first author, year of publication, original literature source, study period, title, cases, average age, age range, histological type, clinical stage, macrophage markers, detection method, TAM distribution, lymph node metastasis and histological grading.

### Quality assessment

NOS was performed for quality assessment on all studies. NOS included study object selection (four items), group comparability (one item) and results measurement (three items) with a total of 9 points. The higher the score, the better the quality. Since the articles in this meta-analysis were all case–control research, Cochrane reviews were also used for quality assessment to evaluate bias of including studies.

### Statistical analysis

On the one hand, IBM SPSS 26 and GraphPad Prism 8 were used in the analysis of experimental data. All *P* values were two-sided and below 0.05 was considered statistically significant. Independent samples *t*-test or ANOVA test was used, and Kruskal–Wallis test was used when unequal variances. And Pearson’s chi-square test was used to assess the association between categorical variables. On the other hand, the software Review Manager 5.0 was used to evaluate the association between tumor-associated macrophage and clinicopathological features of cervical cancer. The software StatTools was used to combine means and standard deviations of two groups into one group. The odds ratio (OR) was used as the effective quantity in the counting data, while the weighted mean difference (WMD) was used as it in the measurement data. The results are expressed in terms of each effect quantity and its 95% confidence interval (CI). The heterogeneity of the included studies was tested by *Q* test.* I*-square (*I*^2^) test was applied to assess total variation among the studies. If *P*-value < 0.10 or *I*^2^ > 50%, the random effect model was applied to pool the data, and the difference was statistically significant (*P*<0.05); otherwise, we chose the fixed-effect model.

## Results

### CD68^+^ and CD163^+^ macrophage infiltration in CC

**Figure 1:**
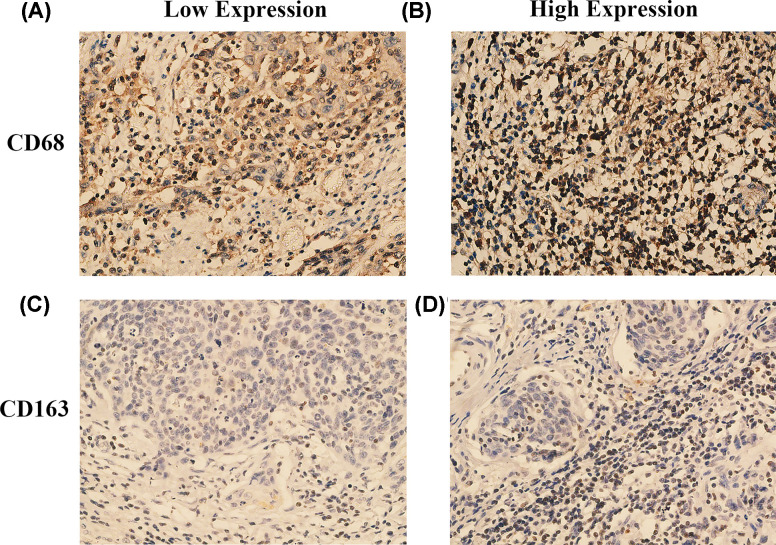
Immunohistochemical staining of CD68 and CD163 expression in CC (**A**) low expression of CD68^+^ TAMs; (**B**) high expression of CD68^+^ TAMs; (**C**) low expression of CD163^+^ TAMs; (**D**) high expression of CD163^+^ TAMs.

**Figure 2:**
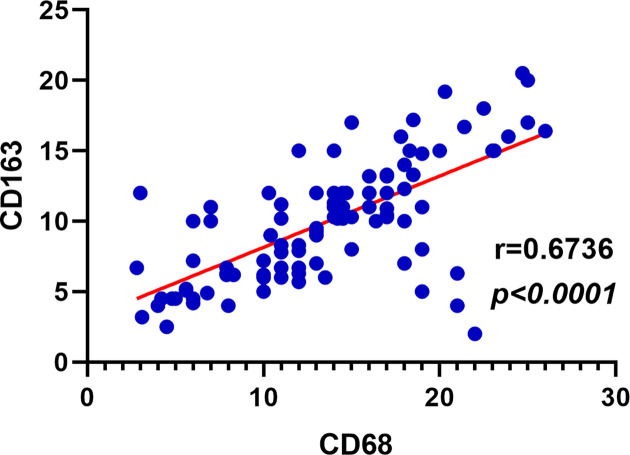
Correlation of CD68^+^ and CD163^+^ macrophages in cervical cancer

We performed immunohistochemical staining for CD68 and CD163 in 100 tissue samples of CC. The CD163 staining pattern showed that the less nonspecific staining of carcinoma cells and other inflammatory factors in a cleaner background than CD68 (Figure 1). The intratumoral density of CD68- positive cells in 100 CC samples was distributed as follows: mean: 13.76; median: 14; standard deviation: 5.63; range: 2.8–26 (Table 1), while the distribution of intratumoral density of CD163- positive cells was as follows: mean: 10.08; median: 10.2; standard deviation: 4.22; range: 2.0–20.5 (Table 1). Although the average number of CD68^+^ macrophages was higher than that of CD163^+^ macrophages, the level of CD68^+^ macrophages showed a positive correlation with CD163^+^ macrophages (*r* = 0.6736, *P*<0.0001, Pearson’s correlation coefficient) (Figure 2).

**Table 1:** Density of CD68- and CD163- positive cells in the tumor field and clinical features in 100 samples of CC

Clinical or pathologic feature	Total number	CD68		CD163	
		CD68 (%) (mean ± SE)	*P-*value	CD163 (%) (mean ± SE)	*P-*value
All cases	100	13.76 ± 5.63	–	10.08 ± 4.22	–
Age at diagnosis			0.27		0.35
<45 years old	32	13.21 ± 7.24		9.62 ± 6.35	
≥45 years old	68	14.02 ± 6.21		10.30 ± 5.21	
Ethnicity			0.46		0.49
Uygur	58	14.13 ± 6.98		10.51 ± 5.94	
Han	32	13.06 ± 7.18		9.27 ± 6.73	
Other minorities	10	13.85 ± 7.23		10.18 ± 7.22	
Childbearing history			0.68		0.54
0–2 times	59	13.89 ± 6.01		10.24 ± 6.31	
≥3 times	41	13.57 ± 6.42		9.84 ± 6.89	
Abortion history			0.56		0.38
Yes	61	13.61 ± 6.32		10.52 ± 5.76	
No	39	13.99 ± 7.89		9.39 ± 6.13	
HPV status			0.59		0.22
HPV 16	34	14.18 ± 6.91		11.05 ± 6.63	
HVP18	38	13.71 ± 6.28		9.36 ± 6.09	
Other	20	13.27 ± 7.67		9.95 ± 7.51	
Negative	8	13.44 ±8.21		9.72 ± 7.89	
Tumor size			0.22		0.64
≤4 cm	47	13.41 ± 6.97		9.95 ± 6.28	
>4 cm	53	14.07 ± 6.45		10.19 ± 5.35	
Differentiation			0.18		0.21
Low	49	13.26 ± 7.02		10.09 ± 5.42	
Middle/High	51	14.24 ± 6.82		10.07 ± 5.29	
FIGO stage			0.19		0.04
IA ∼ IB	52	13.25 ± 6.98		9.07 ± 6.17	
≥ IIA	48	14.31 ± 7.34		11.17 ± 6.42	
Lymph node metastases			0.03		0.04
Yes	32	15.92 ± 8.24		11.23 ± 7.26	
No	68	12.74 ± 6.99		9.54 ± 6.14	

**Abbreviations:** SE, Standard error.

**Note:** Independent-samples *t*-test or ANOVA test was used; Kruskal–Wallis test (K-W) was used when unequal variances. *P*<0.05 was considered significant.

To investigate the clinical significance of CD163^+^ and CD68^+^ macrophages levels in CC, we evaluated the correlation between the levels of CD163^+^ and CD68^+^ macrophages and the clinicopathological features in serial sections of the same tissues. As shown in Table 1, CD163^+^ and CD68^+^ macrophages were not significantly correlated with age, ethnicity, HPV status or differentiation (all *P*>0.05). The counts of total CD163^+^ macrophages were significantly increased in cases presenting with higher FIGO stages (≥IIA group) and lymph node metastasis (both *P*=0.04). The counts of total CD68^+^ macrophages were significantly increased in cases presenting with lymph node metastasis (*P*=0.03). However, there was no significant difference between the expression of CD68^+^ macrophage and FIGO stage.

### Literature searching

**Figure 3:**
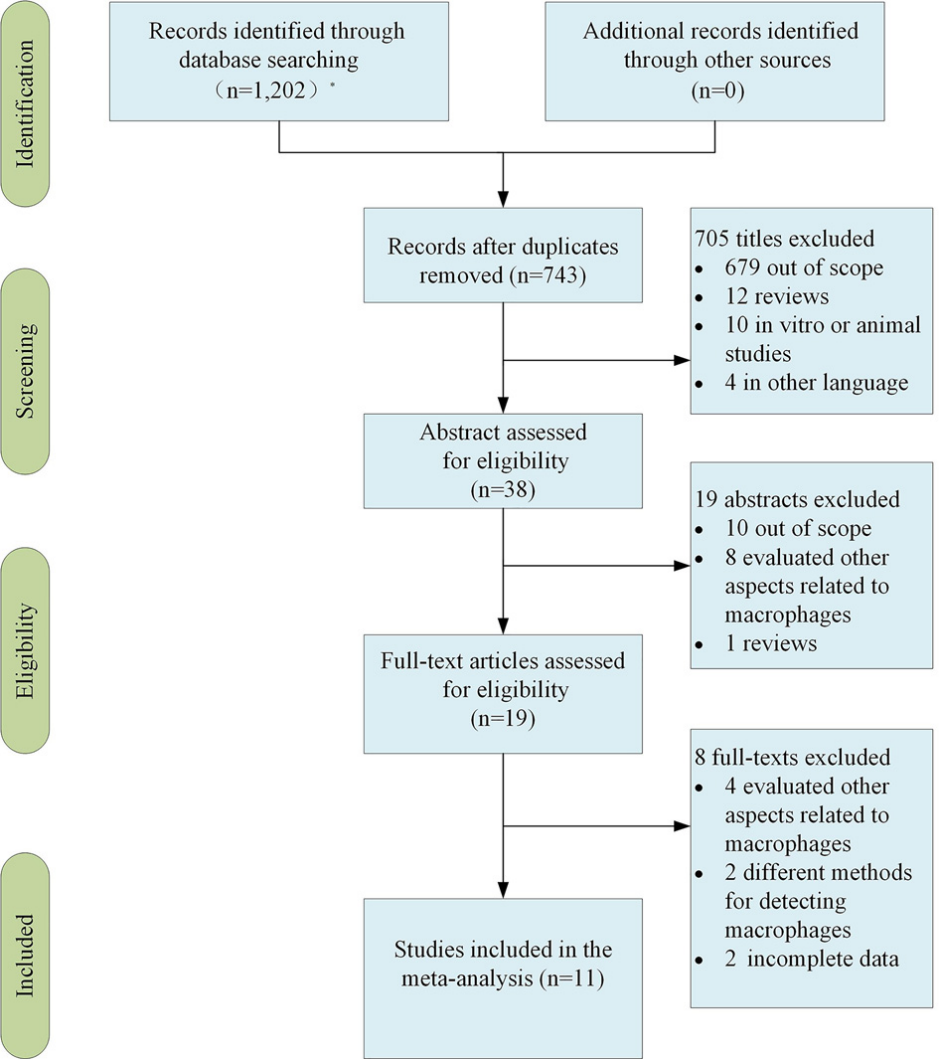
The flowchart showed the different steps in selecting articles included in the systematic review, at last 11 studies were identified for analysis *The database retrieved and the number of checked documents was as follows: PubMed (*n*=515), The Cochrane Library (*n*=0), EMbase (*n*=42), Web of Science (*n*=340), CNKI (*n*=81) and WanFang Data (*n*=224).

A total of 1202 records were identified from different databases during primary search. Out of this total, 459 were duplicate articles. Of the remaining 743 articles, 705 were removed during the title analysis phase: 679 were outside the scope of the research, 12 were reviews, 10 were *in vitro* or animal studies, and another 6 were written in different languages. Another 19 studies were declassified by the abstract evaluation. After these two steps, it remained 19 works in which the full text were analyzed. At present, we discarded eight papers due to the analysis of other aspects, different methods and incomplete data related to macrophages in cervical cancer. At the end, 11 studies [15–25] were included in this review (Figure 3).

### Characteristics of the included studies

The 11 original reports [15–25] published from 2005 to 2019. Detailed information of them was listed in Table 2. All studies applied immunohistochemical staining for TAM markers. CD163 is one of the major markers for recognition of M2 TAMs, and CD68 is a common marker for identifying overall macrophages [10,11]. There were six publications on CD68^+^ and another eight articles on CD163^+^ TAMs, including three studies that estimated both CD68^+^ TAMs and CD163^+^ M2 TAMs simultaneously. The density of TAMs in tumor stroma was studied in nine articles, and the region of TAM density was not mentioned in two articles. In all literatures, the correlation between the number of tumor-associated macrophages and the clinicopathological features of cervical cancer was studied. The results of six articles showed that the high expression of CD68 and CD163 TAMs in tumor stroma or nest was correlated with the clinicopathological features of cervical cancer. And six studies showed that the count of CD68 and CD163 TAMs in tumor stroma or nest was related to the occurrence and development of cervical cancer.

**Table 2:** Characteristics of include studies

Authors/Year	Year	Study period	Age mean (range)	Cases	Type	Markers	Methods	TAM distribution			NOS score
								Stroma	High expression	Counts	
Chen, X.J. et al. [15]	2019	2011–2013	NR	38	SCC	CD163	IHC	✓	_	_	7
Chen, et al. [16]	2019	2012.1–2013.12	NR	72	NR	CD163	IHC	✓	✓	_	6
Yan, et al. [17]	2018	2011.1–2013.12	NR	41	SCC	CD163	IHC	✓	✓	✓	7
Liu, et al. [18]	2018	2013.6–2016.6	50 (35–82)	65	SCC	CD68	IHC	✓	✓	_	7
Wang, et al. [19]	2018	2014.12–2017.6	NR (24–81)	100	SCC, AC and ASC	CD68, CD163	IHC	_	✓	_	6
Li, et al. [20]	2017	2012.1–2012.12	NR	109	SCC	CD163	IHC	✓	_	✓	6
Chen, et al. [21]	2017	2011–2013	NR	45	SCC	CD68, CD163	IHC	✓	✓	✓	7
Shen, et al. [22]	2016	2013–2015	NR (28–71)	60	SCC,AC, ASC and NC	CD163	IHC	✓	_	✓	7
Petrillo, et al. [23]	2015	2009.3–2011.12	55 (22–79)	84	SCC, AC	CD68, CD163	IHC	_	✓	_	6
Ding, et al. [24]	2014	2015.1–2017.12	NR	55	SCC	CD68	IHC	✓	_	✓	7
Liu, et al. [25]	2005	1996.1–1998.12	39 (25–68)	59	SCC, AC	CD68	IHC	✓	_	✓	6

Abbreviations: AC, cervical adenocarcinoma; ASC, adenosquamous carcinoma of cervix; FCM, flow cytometry; IHC, immunohistochemistry; NC, neuroendocrine carcinoma; NR, not reported; SCC, cervical squamous cell carcinoma.

### Quality assessment

**Figure 4:**
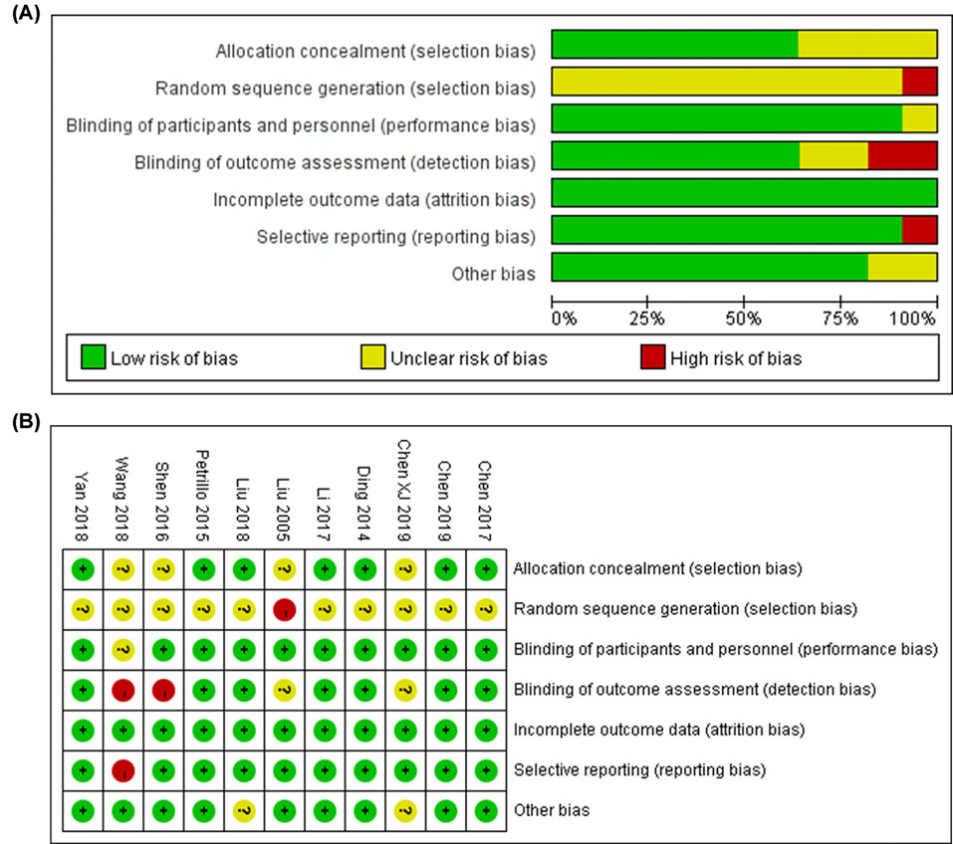
Cochrane reviews for the eligible studies (**A**) Risk of bias graph; (**B**) Risk of bias summary.

The results about quality evaluation of the included studies are shown in Table 2. NOS was performed for quality assessment on all studies. The mean NOS score was 6.54. The detailed score is in Supplementary Table S1. All the quantified studies were the low risk of bias (performance bias and attrition bias) and/or unclear bias for not mentioning the selection and blinded methods in the Cochrane reviews for the eligible ones (Figure 4). As shown in the Figure 4, three articles [19,22,25] which involved selection, detection and reporting bias were considered the high risk of bias. As a whole, the results signified a relatively high level of research selection.

### CD68^+^ TAM expression in cervical cancer

**Figure 5:**
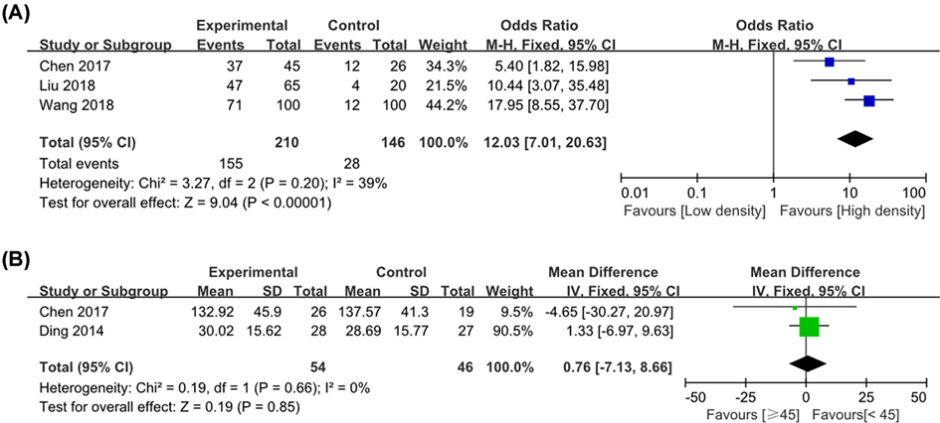
Forest plot of pooled OR and WMD assessed CD68^+^ TAM expression in cervical cancer (**A**) CD68^+^ high expression; (**B**) CD68^+^ TAM number and age of patients.

A total of three publications analyzed the high expression of CD68^+^ TAM in cervical cancer and paracarcinoma or normal tissue. Because there was no statistical heterogeneity between the two groups (*P* = 0.20, >0.10, *I*^2^ = 39%), a fixed-effect model was used. It showed that there was a significant difference between cervical cancer and paracarcinoma or normal tissue (OR = 12.03, 95% CI: 7.01–20.63, *P*<0.001), indicating that the density of CD68^+^ TAM in cervical cancer was much enhanced than that in paracarcinoma or normal tissue (Figure 5A). Two literatures reported the correlation between the total density of CD68^+^ macrophage cell number in tumor stroma and age of patients. It showed that there was no significant difference between CD68^+^ macrophage cell number in tumor stroma and age (≥45 vs. <45 years old, *P*=0.85) (Figure 5B).

### Relationship between CD68^+^ TAMs and clinicopathological parameters of cervical cancer

#### CD68^+^ TAM number and lymph node metastasis

**Figure 6:**
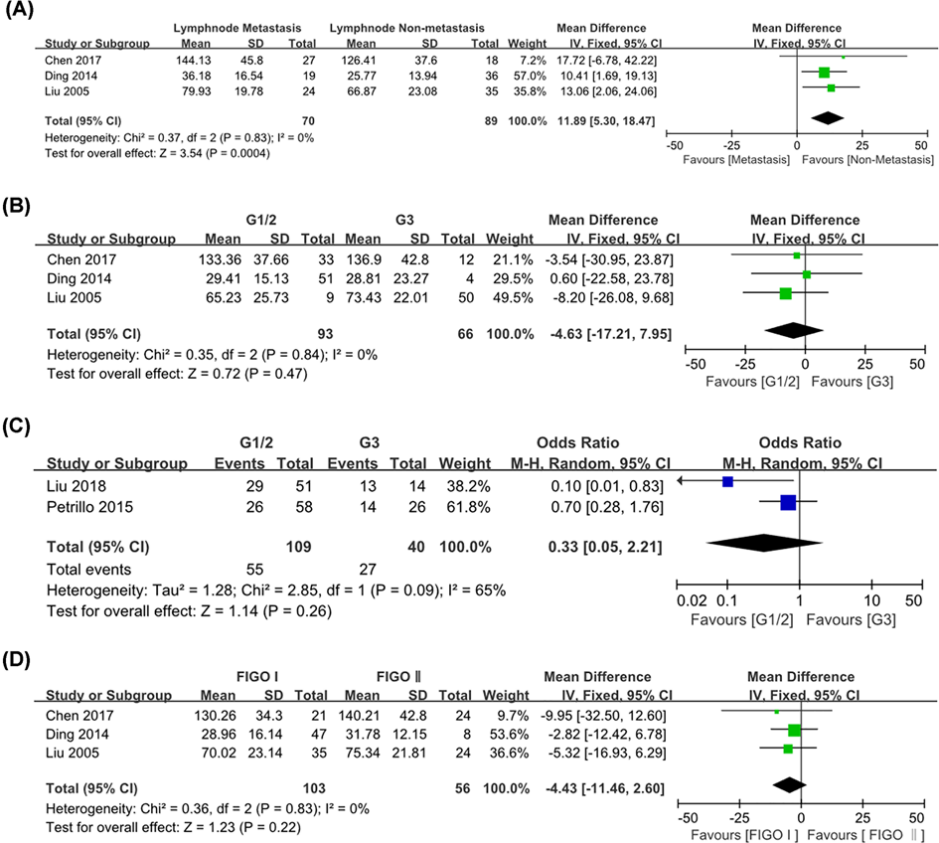
Forest plot of pooled OR and WMD assessed between high CD68^+^ TAM density and clinicopathological features (**A**) CD68^+^ TAM number and lymph node metastasis; (**B**) CD68^+^ TAM number and histological grade; (**C**) CD68^+^ TAM high expression and histological grade; (**D**) CD68^+^ TAM number and FIGO stage.

A total of three studies were included, of which 70 cases had lymph node metastasis and 89 cases had no lymph node metastasis. There was no heterogeneity between the studies (*P*=0.83, *I*^2^ = 0), and the fixed-effect model was used. The results showed that the difference in the total density of CD68^+^ macrophage cell number between the two groups was statistically significant (WMD = 11.89, 95% CI = 5.30–18.47, *P*<0.001) (Figure 6A). It indicated that the density of CD68^+^ TAMs in the tumor stroma with lymph node metastasis was higher than that without lymph node metastasis.

#### CD68^+^ TAMs and different histological grades of cervical cancer

A total of 5 literatures were selected, including 202 cases of high/medium differentiation and 106 cases of low differentiation. On the one hand, there was no significant heterogeneity between the two groups for CD68^+^ TAMs number and differentiation (*P*=0.84, *I*^2^ = 0), so the fixed-effect model was used. On the other hand, the research results showed medium degree of heterogeneity for expression and differentiation (*P*=0.09, *I*^2^ = 65%), then meta-analysis was carried out using a random effect model. The results showed that there were no statistical significance [WMD = -4.63, 95% CI: (-17.21, 7.95), *P*=0.47]; [OR = 0.33, 95% CI: (0.05–2.21), *P*=0.26], indicating that there was no significant difference in the correlation between CD68^+^ TAMs and different histological grades of cervical cancer (Figure 6B,C).

#### CD68^+^ TAM number and FIGO stage

A total of 3 studies were enrolled, including 103 cases of clinical stage I and 56 cases of clinical stage II. The results of the research showed no heterogeneity (*P*=0.83, *I*^2^ = 0%), then meta-analysis was carried out using the fixed-effect model. The results showed that there was no significant difference in the count of stromal CD68^+^ TAMs between the two groups [WMD = -4.43, 95% CI: (-11.46, 2.60), *P*=0.22] (Figure 6D).

### CD163^+^ M2 TAM density in cervical cancer

**Figure 7:**
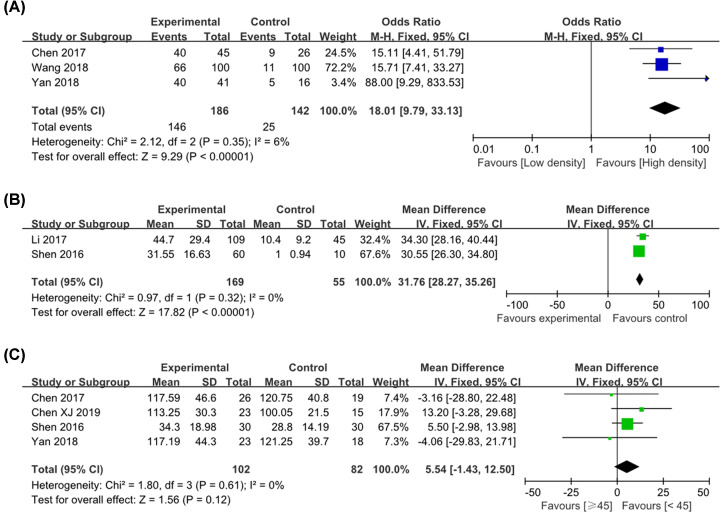
Forest plot of pooled OR and WMD assessed CD163^+^ M2 TAM expression in cervical cancer (**A**) CD163^+^ high expression; (**B**) CD163^+^ M2 TAM number; (**C**) M2 TAM number and age of patients.

On the one hand, three publications reported CD163^+^ TAM density in cervical cancer and paracarcinoma or normal tissue. Since there was no statistical heterogeneity between the two groups (*P*=0.35, *I*^2^ = 6%), a fixed-effects model was used. It showed that there was significant difference between cervical cancer and paracarcinoma or normal tissue (OR = 18.01, 95% CI = 9.79–33.13, *P*<0.001), indicating that M2 TAM density in cervical cancer was much enhanced than that in paracarcinoma or normal tissue (Figure 7A). On the other hand, the two groups used quantitative data to analyze the number of CD163^+^ TAM between cervical cancer and paracarcinoma or normal tissue. The results also showed that there was a difference between the two groups [WMD = 31.76, 95% CI: (28.27–35.26), *P*=0.32; *I*^2^ = 0%, *P* < 0.001; Figure 7B]. Besides, four literatures analyzed the correlation between the total density of M2 macrophage cell number in tumor stroma and age of patients. However, there was no association between the density of CD163^+^ TAMs in the stroma and age (≥45 vs. < 45 years old) [WMD = 5.54, 95% CI: (-1.43, 12.50), *P*=0.12; *I*^2^ = 0%, *P*=0.61; Figure 7C].

### Relationship between CD163^+^ M2 TAM infiltration and clinicopathological characteristics of cervical cancer

**Figure 8:**
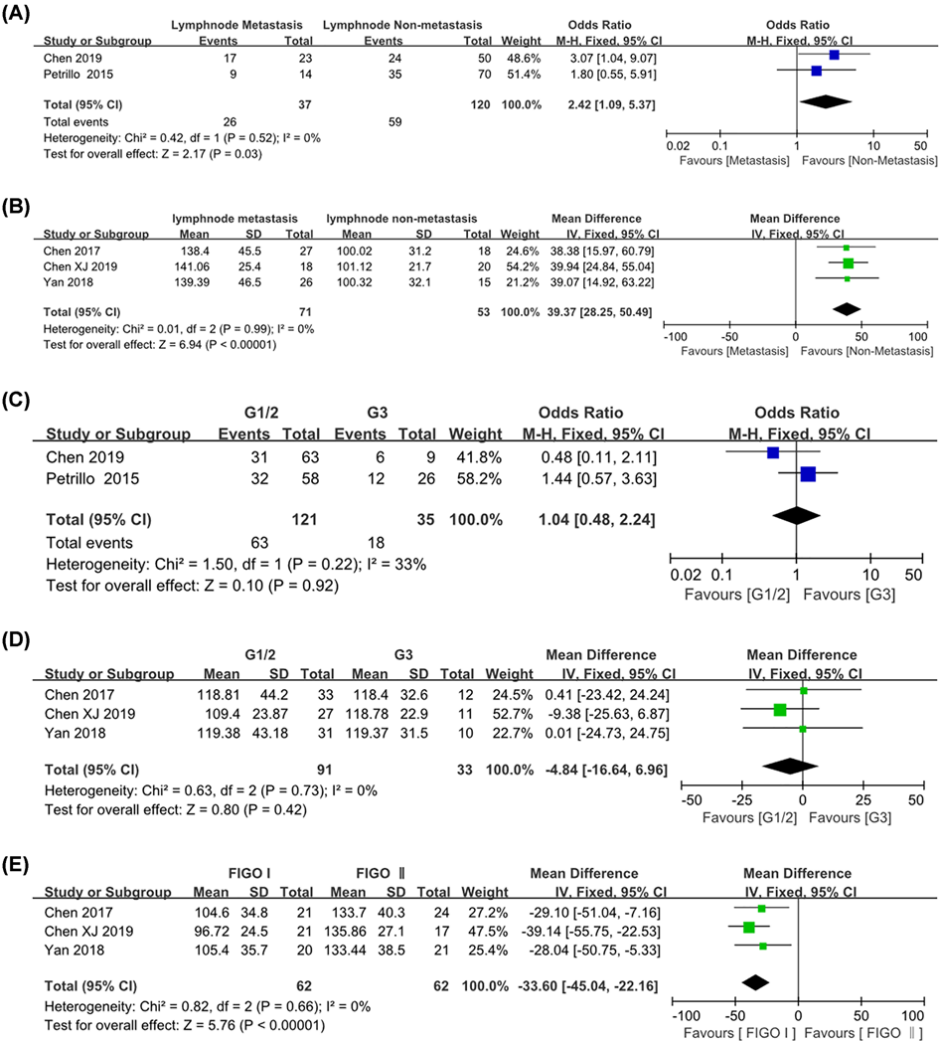
Forest plot of pooled OR and WMD assessed between CD163^+^ TAM density and clinicopathological features (**A**) CD163^+^ high expression and lymphnode metastasis; (**B**) CD163^+^ TAM number and lymph node metastasis; (**C**) CD163^+^ TAM high expression and histological grade; (**D**) CD163^+^ TAM number and differentiation; (**E**) CD163^+^ TAM high expression and FIGO stage.

Some of the literatures reported the association between M2 TAM density and clinicopathological characteristics. We focused on the association between CD163^+^ M2 TAM infiltration and clinicopathological parameters of cervical cancer, such as lymph node metastasis, histological grade as well as FIGO stage. As for CD163^+^ M2 TAMs, high stromal CD163^+^ TAM density was associated with lymph node metastasis [OR = 2.42, 95% CI: (1.09, 5.37), *P*=0.03; *I*^2^ = 0%, *P*=0.52]; [WMD = 39.37, 95% CI: (28.25, 50.49), *P*<0.001; *I*^2^ = 0%, *P*=0.99]. They were shown in Figure 8A,B separately. However, there was no difference between different histological types (G1/2 and G3) and high M2 TAM density groups [OR = 1.04, 95% CI: (0.48, 2.24), *P*=0.92; *I*^2^ = 33%, *P*=0.22] (Figure 8C). And there was no significant difference in correlation between the number of CD163^+^ TAM and different histological grades of cervical cancer [WMD = -4.84, 95% CI: (-16.64, 6.96), *P*=0.42; *I*^2^ = 0%, *P*=0.73] (Figure 8D). They were shown separately in Figure 8C,D. Surprisingly, high stromal CD163^+^ TAM number was associated with FIGO stage [WMD = -33.60, 95% CI: (-45.04, -22.16), *P*<0.001; *I*^2^ = 0%, *P*=0.66] (Figure 8E).

### Publication bias

**Figure 9:**
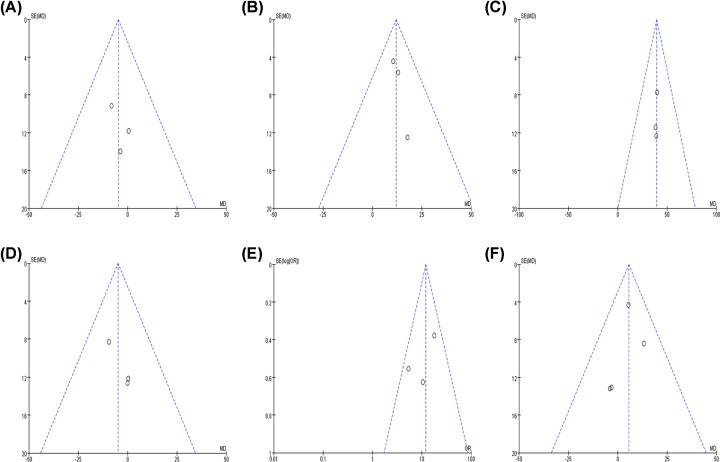
Funnel plots for detection of publication bias The pseudo 95% confidence interval (CI) is computed as part of the analysis that produces the funnel plot and corresponding to the expected 95% CI for a given standard error (SE). (**A**) CD68^+^ TAM number and differentiation; (**B**) CD68^+^ TAM number and lymph node metastasis; (**C**) CD163^+^ TAM number and lymph node metastasis; (**D**) CD163^+^ TAM number and differentiation; (**E**) CD68^+^ TAM high expression; (**F**) CD163^+^ TAM number and age of patients.

Funnel chart analysis showed that the included studies had no obvious publication bias by visual inspection (Figure 9). Due to the limited number of enrolled literatures, we abandoned the evaluation of the publication bias of Egger’s and Begg’s test.

## Discussion

CC is the second most common malignant disease among women in the whole world. The latest cancer data in 2021 in the United States nationally report that women have the fourth highest incidence of cervical cancer and the eighth highest mortality rate among cancer deaths [26]. Traditional treatments for CC included surgery, radiotherapy and chemotherapy. Immunotherapy, an emerging method of tumor treatment, is being popularized in clinic. TME is a complex system, and it is defined as a complex environment that supports cancer progression, proliferation of tumor cells and invasion of adjacent tissues. Recent studies have highlighted the important role of TME in the progression, invasion and metastasis of CC [27]. A comprehensive understanding of TME can provide research directions for finding novel immunotherapeutic agents. TAMs are a critical component of TME, accounting for 30–50% of the TME cells. A key regulator of tumor immunity is the TAM population. They promote tumor progression through various mechanisms, including therapeutic resistance, intravascular perfusion, angiogenesis, immune suppression and metastasis [28]. The multifunctional characteristics of TAMs in tumor progression suggest that targeting this group of immune cells may represent a new immunotherapeutic strategy.

In the process of tumorigenesis and development, many proinflammatory mediators recruit circulating monocytes into tumor, inflammatory or infected tissues and obtain the characteristics of infiltrating macrophages. In tumor tissue, TAMs mainly show two types of properties, namely ‘M1-like’ and ‘M2-like’. These properties are affected by various growth factors, metabolic needs, local oxygen tension, tissue cells and tissue matrix [29]. As a matter of fact, monocyte-derived macrophages in tissues are usually highly heterogeneous when they experience a variety of influencing factors. For example, four subsets of M2 have been identified, including M2a, M2b, M2c and M2d [30].

‘M1-like’ macrophage polarization mainly occurs in the presence of interferon gamma (IFN-γ) or exposure to microorganisms or their products such as lipopolysaccharide (LPS) [31]. Then, M1 secretes several proinflammatory cytokines such as IL-1 and IL-6, which are associated with activation of Th1 response and Th1 lymphocytes attraction [32]. Moreover, ‘M1-like’ macrophages can phagocytose and kill target cells [33]. ‘M2-like’ macrophage polarization is stimulated in response to IL- 4 or IL-13 [34], expressing abundant scavenger receptors and is associated with high production of IL-10, IL-1b, vascular endothelial growth factor (VEGF) and matrix metalloproteinases (MMP).

In the process of cervical inflammation, the phenotype and function of TAMs are constantly changing, which are involved in different regulatory networks. TAMs type conversion plays a non-negligible role in tumor invasion and metastasis. In the CC microenvironment, the transformation of TAMs belongs to transformation of the immune type, which plays an important role in the prognosis of CC [35]. For the mixed phenotype of macrophages in the microenvironment, recent studies have shown that TAMs have functions similar to those of M2-like macrophages [36]. Therefore, our study also focused on evaluating the role of M2 TAMs in the occurrence and development of cervical cancer.

Previous research have reported that macrophages are mainly stained with CD68. However, CD68 is a molecular marker of pan-macrophages, which cannot distinguish different phenotypes of macrophages infiltrated in tumor tissues. CD68 and CD163 were detected at the same time in our immunohistochemical experiment. In the meta-analysis, a total of three out of the eleven included studies [18,24,25] used CD68 as macrophage marker, while the other three studies [19,21,23] used CD68 combined with CD163 to detect TAMs. CD163 served as a specific marker for TAMs with the M2 phenotype [37]. A total of five [15–17,20,22] out of the eleven studies individually used CD163 as macrophage marker.

In the included studies, the density of CD68^+^ and CD163^+^ TAM in cervical cancer was significantly enhanced than those in paracarcinoma or normal tissue. There was no relationship between the CD68^+^ TAM density in cervical cancer and clinicopathological features, including age, histological grades and clinical stage. And there was no association between the stromal CD163^+^ TAMs density and clinicopathological features, such as age and histological grades. However, high stromal CD68^+^ TAMs density was relevant to lymph node metastasis. Furthermore, high stromal CD163^+^ M2 TAM infiltration was found to be associated with more advanced FIGO stage. Both CD68^+^ and stromal CD163^+^ M2 TAM density were associated with lymph node metastasis in CC. However, as can be seen from the results of this meta-analysis, CD68^+^ TAM and CD163^+^ M2 TAM density in cervical cancer were significantly enhanced than those in paracarcinoma or normal tissue. And M2 TAM infiltration was associated with more advanced FIGO stage and lymph node metastasis. These findings indicated that the number of TAM infiltration was correlated with disease progression in CC. This was confirmed in our 100 experimental studies of CC. In our study, the intratumoral density of CD163- positive cells was significantly higher in FIGO stage (≥ IIA group) than in FIGO stage (IA ∼ IB group) (*P*<0.05). And the intratumoral density of CD68- and CD163- positive cells in cervical carcinoma with lymph node metastasis was significantly higher than that in non-lymph node metastasis group (both *P*<0.05).

This was consistent with some studies mentioned in a review [38]. They suggested that a high number of tumor-associated macrophages were beneficial for tumor growth and associated with disease progression and poor prognoses for the patients. However, sometimes a high number of infiltrating TAMs were correlated with better prognosis. For instance, in cervical intraepithelial neoplasia induced by human papilloma virus (HPV), TAM infiltration was found to be correlated with disease progression [39]. Yet, the number of stromal TAMs was positively related to the intratumoral expression of IL-12p40 in cervical carcinoma, and IL-12p40 itself was associated with a favorable overall survival (OS) in patients with CC [40]. Due to the limited data available, the stratified analysis was not performed to evaluate the correlation between infiltrating TAMs and prognoses.

Meta-analysis is essentially an observational study. After systematically analyzing and evaluating the existing literatures related to CC and M2 TAMs infiltration from the point of view of evidence-based medicine, we also used immunohistochemical technique to detect the expression of M2 TAMs marker in CC, and further discussed the clinicopathological significance of M2 TAMs infiltration in CC. Though we tried our best to perform a comprehensive analysis between CD68^+^ and CD163^+^ TAM infiltration and clinicopathological features in CC, there were still some limitations of the present study. First, although CD163 was used as a molecular marker of M2 TAMs to detect the invasion of M2 TAMs in CC, there was no clear report on the markers of M1 macrophage. Second, the number of samples included in the study was relatively small and the sample size was relatively insufficient. Third, there was a potential risk of publication bias since we only included the publications in English and Chinese. And lack of gray literature may also result in missing negative results. Fourth, in the assessment of positive expression of CD68 and CD163 TAMs, Liu et al. [18] showed that scores were based on the intensity of TAMs staining and the number of cells. Comprehensive evaluation of the number of positive cells ≤25% was low expression, >25 was high expression. Chen et al. [21] and Yan et al. [17] assessed the percentage of positive cells <20% for low expression and ≥20 for high expression were based on the proportion of positively stained cells. These studies have different threshold values when assessing TAM expression to distinguish between high and low groups, i.e. patients with CD68^+^ TAM infiltration in the study do not necessarily belong to the same group. Patients with a low CD68^+^ TAM count in one study could be divided into high CD68^+^ TAM groups in another study due to different cut-off values. This makes the results of this study may be biased. Last, the main research object of the present study was paraffin section of tumor tissue, paracarcinoma and normal tissue. However, IHC method may have certain technical bias affected by reagent, evaluation methods of positive result, skill of the operators and so on. In order to provide stronger evidence for evidence-based medicine in the progress of CC, more rigorous and high quality case–control studies with standardized methodology should be carried out to further confirm the correlation between the expression of TAM infiltration and clinicopathological features of CC.

In summary, the present study confirmed that the infiltration of CD68^+^ TAM and CD163^+^ M2 TAM had obvious difference in tumor tissue of CC and normal cervical tissue. Their expressions were significantly related to the lymph node metastasis, and there was no significant difference in the patients’ age and histological grades. Furthermore, high stromal CD163^+^ M2 TAM infiltration was found to be associated with more advanced FIGO stage. Therefore, TAMs may be associated with the entire occurrence, invasion and metastasis of CC. High stromal M2 TAMs also showed positive association with tumor progression, which may indicate a poor prognosis.

## Supplementary Material

Supplementary Table S1

## Data Availability

Data supporting this study are available within the manuscript file.

## Abbreviations

AC: cervical adenocarcinoma
ASC: adenosquamous carcinoma of cervix
CC: cervical carcinoma
CI: confidence interval
FCM: flow cytometry
FIGO: International Federation of Obstetrics and Gynecology
HPV: human papilloma virus
I^2^: *I*-square
IFN-γ: interferon gamma
IHC: immunohistochemistry
LPS: lipopolysaccharide
MMP: matrix metalloproteinase
NC: neuroendocrine carcinoma
NOS: Newcastle–Ottawa scale
NR: not reported
OR: odds ratio
OS: overall survival
PBS: phosphate buffer saline
SCC: cervical squamous cell carcinoma
SE: standard error
TAM: tumor-associated macrophage
TME: tumor microenvironment
VEGF: vascular endothelial growth factor
WMD: weighted mean difference
