# Supplementary material for: The correlation between tumor-associated macrophage infiltration and progression in cervical carcinoma
Source: Biosci Rep. 2021 May 20;41(5):BSR20203145. doi: 10.1042/BSR20203145 (PMC8493445; doi:10.1042/BSR20203145)
Supplement: Supplementary Table S1 [file BSR-2020-3145_supp.pdf]

Supplementary Table 1 Results of the evaluation of the risk of bias in the included studies

**Studies**	**Appropriateness of the case (1 point)**	**Representation of the case (1 point)**	**Selection of the control (1 point)**	**Determination of the control (1 point)**	**Comparability of the case and the control in the design and statistical analysis (2 points)**	**Exposure factors (1 point)**	**The same method to determine exposure factors of the case and contro(1 point)**	**No response rate (1 point)**	**NOS（9 point)**
Chen XJ et al [15]	1	1	0	1	2	1	1	0	7
Chen et al [16]	1	1	0	1	1	1	1	0	6
Yan et al [17]	1	1	0	1	2	1	1	0	7
Liu et al [18]	1	1	0	1	2	1	1	0	7
Wang et al [19]	1	1	0	1	1	1	1	0	6
Li et al [20]	1	1	0	1	1	1	1	0	6
Chen et al [21]	1	1	0	1	2	1	1	0	7
Shen et al [22]	1	1	0	1	2	1	1	0	7
Petrilloet al l [23]	1	1	0	1	1	1	1	0	6
Ding et al [24]	1	1	0	1	2	1	1	0	7
Liu et al [25]	1	1	0	1	1	1	1	0	6
